# Evaluation of HER2 and p53 expression in predicting response to docetaxel-based first-line chemotherapy in advanced breast cancer

**DOI:** 10.1186/1756-9966-30-38

**Published:** 2011-04-11

**Authors:** Andrea Camerini, Sara Donati, Paolo Viacava, Olimpia Siclari, Cheti Puccetti, Gianna Tartarelli, Chiara Valsuani, Filomena De Luca, Leonardo Martini, Andrea Cavazzana, Domenico Amoroso

**Affiliations:** 1 Oncology Department, Medical Oncology Division, AUSL12 di Viareggio and Istituto Toscano Tumori - Versilia Hospital, Lido di Camaiore, Italy; 2 Oncology Department, Pathology Division, AUSL12 di Viareggio and Istituto Toscano Tumori - Versilia Hospital, Lido di Camaiore, Italy; 3 Pathology Division, AUSL 1 Massa-Carrara and Istituto Toscano Tumori, Carrara, Italy

## Abstract

**Background:**

The human epidermal growth factor receptor 2 (HER2) and p53 pathways may be involved in chemotherapy sensitivity and/or resistance. We explore the value of HER2 and p53 status to foretell docetaxel sensitivity in advanced breast cancer.

**Methods:**

HER2 and p53 expression was analysed in 36 (median age 55 yrs; range 37-87) metastatic breast cancer patients receiving docetaxel-based first-line chemotherapy. HER2 was determined by immunohistochemistry (IHC) and fluorescence *in situ *hybridization (FISH), p53 was tested by IHC. We correlate the expression of study parameters with pathologic parameters, RECIST response and survival. The standard cut-off value of 2 was used to determine HER2 overexpression while p53 mean expression level was used to divide low/high expressors tumors.

**Results:**

Median time to progression and overall survival were 9 (range 2 - 54) and 20 (range 3 - 101) months. Overall response rate was 41.6%. Nine cases showed HER2 overexpression. HER2 was more frequently overexpressed in less differentiated (*p *= 0.05) and higher stage (*p *= 0.003) disease. Mean FISH-HER2 values were significantly higher in responder than in non-responder pts (8.53 ± 10.21 vs 2.50 ± 4.12, *p *= 0.027). Moreover, HER2 overexpression correlates with treatment response at cross-tabulation analysis (*p *= 0.046). p53 expression was only associated with higher stage disease (*p *= 0.02) but lack of any significant association with HER status or docetaxel response. No significant relation with survival was observed for any parameter.

**Conclusion:**

Our data seem to indicate that FISH-determined HER2 status but not p53 is associated with docetaxel sensitivity in metastatic breast cancer.

## Background

Breast cancer (BC) is the leading cause of cancer and the second leading cause of cancer death in women in the USA [1] and its incidence is increasing in many countries, including Italy [2] thus representing a major health problem. To date, the role of chemotherapy in BC treatment is certain and taxanes are widely used in both early and advanced setting [3,4] but we have no validated sensitivity and/or resistance predictive factor and, hence, the search for a taxane-specific predictive marker is an hot topic. Colled as the "guardian of the genome" [5] and the "cellular gatekeeper" [6], the p53 protein acts as cell modulator by driving lots of stress-inducing signals to different antiproliferative cellular responses [7]. p53 can be activated in response to DNA damage (such as cytotoxic agents), oncogene activation or hypoxia resulting to cellular outputs such as apoptosis, cell-cycle arrest, senescence, or modulation of autophagy [8-10]. Although about 50% of BCs harbours *TP53 *gene mutations [11,12], the biological role and clinical importance of p53 alterations in BC are still unclear. This maybe related to the very complicated and extensive p53 network and to technical problems associated with surrogate markers to identify *TP53 *gene defects, as most detection tests lack sensitivity and specificity. Despite its limits, immunohistochemical p53 detection demonstrated in numerous studies to be a prognostic factor in BC [11-17] and that it may determine the sensitivity to specific therapeutic agents [18-22]. Some evidences may indicate that abnormal p53 expression could be associated with taxane sensitivity but its specific predictive role is unclear [22-24].

Another leading cell growth regulator in BC is the human epidermal growth factor receptor (HER) 2 (HER2; erbB2/neu). The HER2 oncogene encodes one of four trans-membrane receptors within the erbB family. Its over-expression, which occurs in approximately 25% of all breast cancer tumors, is associated with a shortened disease-free interval and poor survival [25]. HER2 blockage in preclinical models of human BC and in primary breast tumors from women treated with HER2-targeted therapies leads to the inhibition of survival pathways, which in turn induces tumor cell apoptosis [26]. The clinical benefit of HER2 inhibition by its specific monoclonal antibody trastuzumab is meaningful in both early and advanced disease [27,28]. HER2 status may also influence chemotherapy sensitivity as proposed by Gennari et al [29] that focused on the adjuvant setting showing that the added benefits of adjuvant chemotherapy with anthracyclines seems to be reserved to breast cancer harboring HER2 overexpression or amplification.

On this grounds, we analysed the relationship between HER2 and p53 expression and response to first-line docetaxel based chemotherapy in advanced BC finding that FISH-determined HER2 status but not p53 could predict docetaxel sensitivity.

## Methods

### Patient characteristics and tissue samples

Tumor samples were obtained from breast cancer patients who underwent surgery at Versilia Hospital in Lido di Camaiore (Italy) from 2000 to 2004. A total of 36 breast cancer patients (median age 55 yrs; range 37-87) receiving between 2001 and 2005 a docetaxel-based first-line chemotherapeutic regimen for metastatic disease were retrospectively selected for the study. Study population characteristics are shown in table 1. Mean time from initial diagnosis to first relapse was 15.8 ± 6.5 months. Location of metastatic deposits includes bone (21/36), liver (21/36), lung (16/36), lymphnodes (14/36) and local recurrence (3/36) with 27 out of 36 patients presenting with multiple disease sites; remaining 9 patients with single-site metastasis presented with measurable non-bone disease. Patients receiving pre-operative chemotherapy, having a family history of breast cancer or receiving docetaxel as part of adjuvant treatment were excluded as well as those for whom follow-up data were missing. Adjuvant treatment was performed in all patients but two as follow: 18 patients received an association of 5-fluorouracil (5-FU), epirubucin and cyclophosphamides (FEC) for 6 cycles, 11 patients received an association of epirubucin and cyclophosphamides (EC) for 4 cycles, and remaining 5 patients received an association of cyclophosphamides, methotrexate and 5-FU (CMF) for 6 cycles.

**Table 1:** Study population characteristics (n = 36)

Median [range] age (yr)	55 [37-87]
**Histotype^#^**	
Invasive ductal carcinoma	28 (77.7%)
Invasive lobular carcinoma	5 (13.8%)
Mixed (ductal and lobular)	2 (5.5%)
Undifferentiated	1 (3.0%)
**Grading°**	
G2	21 (58.3%)
G3	15 (41.7%)
**ER status**	
Negative	14 (38.8%)
Positive	22 (61.2%)
**PgR status**	
Negative	13 (36.1%)
Positive	23 (63.9%)
HER2 status*	
Negative	27 (75.0%)
Positive	9 (25.0%)
**Adjuvant chemotherapy^**	
FEC	18 (52.9%)
EC	11 (32.4%)
CMF	5 (14.7%)
**Mean ± SD time to first relapse (months)**	15.8 ± 6.5
**Metastatis sites**	
Bone	21 (58.3%)
Liver	21 (58.3%)
Lung	16 (44.4%)
Lymphnodes	14 (38.8%)
Local	3 (8.3%)
**Chemotherapy"**	
TXT75	14 (38.8%)
TXT25	8 (22.2%)
TXT75+C	5 (13.8%)
TXT75+T	9 (25.2%)
**Treatment best response**	
Complete response	1 (2.7%)
Partial response	14 (38.8%)
Stable disease	12 (33.3%)
Disease progression	9 (25.2%)
**Time to disease progression (months)**	
Median [range]	9 [2-54]
**Overall survival (months)**	
Median [range]	20 [3-101]

^#^According to WHO hystological typing of breast tumor (Ref. 32). °According to Elston and Ellis classification (Ref. 31). *Pre-study determination. "See text for regimen details. **^**on 34 pts.

All patients received docetaxel-based first-line chemotherapy for metastatic disease. In particular, 14 out of 36 patients received six cycles docetaxel (75 mg/m^2^) every 3 weeks (TXT75), 8 patients received docetaxel (25 mg/m^2^) on a weekly basis (TXT25), 5 patients received a combination of docetaxel (75 mg/m^2^) on day 1 plus capecitabine (1000 mg/m^2 ^bid day 1-14) every 3 weeks (TXT75+C) and the remaining 9 patients with HER2-positive disease received a combination of docetaxel (75 mg/m^2^) and trastuzumab (8 mg/kg loading dose then 6 mg/kg) both on day 1 every 3 weeks (TXT75+T) (Table 1). Every treatment was planned for up to 6-9 months. Causes for early treatment stop were unacceptable toxicity, disease progression or patient refusal. Trastuzumab was administered alone after docetaxel discontinuance as maintenance therapy until disease progression in 6 responder patients. Tumor assessment was performed every 3 months by CT-scan and/or chest X-ray coupled with abdomen ultrasound depending on those used at baseline. Time to progression (TTP) was calculated from the date of treatment start to the date of first-documented progression. Overall survival (OS) was defined as the time interval between the start of treatment and death or last follow-up contact. Treatment response was assessed according to RECIST criteria and we consider as responder a patient achieving a complete (CR) or partial (PR) response to treatment. Patients achieving disease stabilization (SD) or disease progression (PD) were considered as not-responders. Anyway, we planned a secondary analysis considering as responders even patients achieving disease stabilization as best result. Median TTP was 9 (range 2 - 54) months and overall response rate (ORR) was 41.6% (14 out of 36) with 11 and 8 pts experiencing disease stabilization and progression respectively. Median OS was 20 (range 3 - 101) months. Being a retrospective analysis patients were not asked to sign any informed consent; anyway samples were coded and the names of the patients were not revealed. All available clinico-pathological data were collected and stored in an appropriate database. Age, tumor grade and stage [30,31], size, histotype,(32) estrogen receptor (ER) and progesterone receptor (PgR) status were considered.

### Immunoistochemistry

P53 expression was evaluated by immunohistochemistry (IHC) while HER2 expression was evaluated both by IHC and fluorescence *in situ *hybridization (FISH - see next paragraph). All IHC analyses were performed on routinely processed, formalin-fixed and paraffin-embedded tissue samples obtained from primary tumor. For p53 IHC analysis, representative tumor sections (3 μm) were deparaffinized, rehydrated and immunostained using antigen retrieval by microwave technique. After endogenous peroxidase blocking sections were incubated for 45 min at 37°C with a 1:50 dilution of primary mouse anti-human *p53 *monoclonal antibody (clone: DO-7, isotype IgG2b) (Dako), then immunostained with secondary antibodies and finally counterstained with hematoxylin. Sections of known positive mammary carcinoma were used as positive controls. Negative controls were obtained by omitting the primary antibodies. For p53 only a clear nuclear staining in the absence of cytoplasmic background coloration was considered positive. A minimum of 1.000 cells were counted for each tumor and immunoreactivity was expressed as a percentage of positive cells on the total number of tumor cells. A value of 11% of positive cells, corresponding to the mean value of p53 expressing tumor cells, was used as cut-off to distinguish high and low expressing tumors.

HER2 IHC evaluation was realized by the streptavidin-biotin-peroxidase complex technique (StreptABC, DAKO) as standard for the time of analysis. Tissue sections were deparaffinized and underwent antigenic retrieval and endogenous peroxidase blocking. Sections were first incubated with polyclonal primary antibodies against c-erbB-2 (A0485, DAKO) with a 1:500 dilution, then incubated in secondary biotinylated antibody and finally counterstained with Hematoxylin. Immunohistochemical analyses of c-erbB-2 expression describe the intensity and staining pattern of tumor cells. The FDA-recognized test, the Herceptest™ (DAKO), describes four categories: no staining, or weak staining in fewer than 10% of the tumor cells (0); weak staining in part of the membrane in more than 10% of the tumor cells (1+); complete staining of the membrane with weak or moderate intensity in more than 10% of the neoplastic cells (2+); and strong staining in more than 10% (3+). Cases with 0 or 1+ score were regarded as negative; the ones with 3+ score were regarded as positive while 2+ cases underwent FISH and categorized accordingly. All immunostained specimens were evaluated by two observers independently (PV and AC) without knowledge of clinical characteristics and/or follow-up information and the discrepant cases were jointly re-evaluated and agreement was met.

### Dual-color Fluorescence *in situ *Hybridization

HER2 amplification was analyzed on microdissected tumor samples using FISH HER2 PharmDx (Dako, K5331), which contains both fluorescently-labeled HER2/neu gene and chromosome 17 centromere probes. Microdissection was performed by an expert pathologist different from ones that performed IHC evaluation. In brief, sections were deparaffinized, heat-pretreated in citrate buffer at 80°C for near 1 hour, digested with pepsin at room temperature for few minutes and dehydrated in graded ethanol. After the HER2/CEN17 probe mix was applied to the dry slides. The slides were then incubated in hybridizer (Hybridizer Instrument for *in situ *hybridization, DAKO, S2450) for denaturation at 82°C for 5 minutes and hybridization at 45°C for about 18 hours. The slides were re-dehydrated in graded ethanol. FISH analyses were performed according to the HER2 FISH PharmDx (Dako) criteria. In each case, 100 non-overlapped, intact interphase tumor nuclei identified by DAPI staining were evaluated, and gene (red signal) and CEN17 (green signal) copy numbers in each nucleus were assessed. The cases were considered to be amplified when the average copy number ratio, HER2/CEN17, was ≥ 2.0 in all nuclei evaluated or when the HER2 signals formed a tight gene cluster. Among the cases in which the gene was not amplified, samples showing more than four copies of the HER2 gene and more than four CEN17 in more than 10% of the tumor cells were considered to be polysomic for chromosome 17.

### Statistical analysis

**Correlation **between p53, HER2 and other molecular and clinical parameters were assessed by contingency table methods and tested for significance using the Pearson's chi-square test. Mean values were compared using the student-T test. Survival curves were calculated using the Kaplan-Meier method and tested for significance using the log-rank test. Univariate and multivariate relative risks were calculated using Cox proportional hazards regression. Statistical analyses were performed using NCSS software. All tests were two-tailed, and p < 0.05 was considered to be significant.

## Results

**Figure 1:**
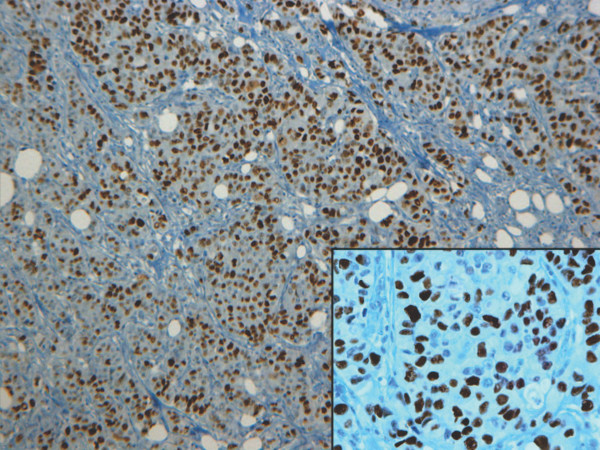
**Immunohistochemical positive staining of p53 in a representative case of high-grade (G3) ductal carcinoma**. Immunostaining shows a clear and wide nuclear staining in an high grade (G3) invasive ductal carcinoma. Original magnifications: ×100 (inset ×250).

**Figure 2:**
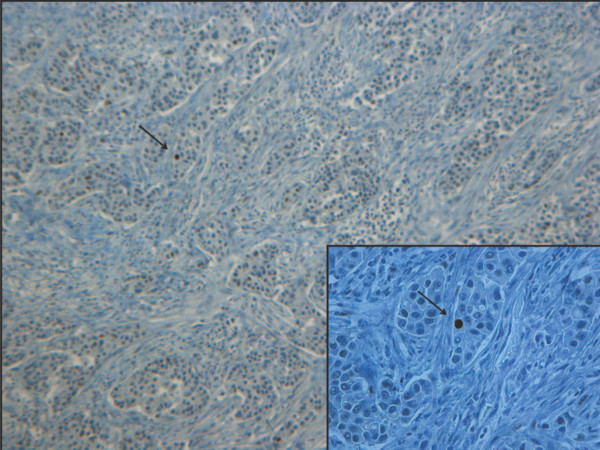
**p53 immunohistochemical negative staining in a grade 2 ductal carcinoma**. The wide majority of nuclei showed no staining with the exception of one clear positive nucleus (arrow) in the upper left corner. Original magnifications: ×100 (inset ×250).

Expression levels of p53 ranged from 0% to 70% of immunostained nuclei with a mean expression value of 11% (median = 5%) (Figure 1 and 2). Using this mean value as cut-off to distinguish high and low expressing tumors, staining was considered high in 11 (30.5%) out of 36 tumors in our series (similar results were obtained using as cut-off the median value). P53 expression levels were only related to disease stage with higher p53 levels in higher stage disease (*p *= 0.02) but lack of any significant association with HER2 status, other clinic-pathologic parameters (age, ER and PgR status, Ki67 and tumor grading) or docetaxel response (Table 2). Even comparing mean p53 expression levels between responders vs not-responders patients we did not find any significant difference (not shown) and mean TTP (8.6 ± 7.0 vs 9.2 ± 11.9 months; *p *= ns) and OS (21.6 ± 13.0 vs 19.8 ± 10.2 months; *p *= ns) did not differ between low and high p53 groups. Morever, no significant relation with survival parameters was observed for p53 at Kaplan-Meier analysis.

**Table 2:** p53 expression in relation to main tumor characteristics and treatment response

	p53 expression			
	
	Total	Low	High	*p *value
**Age**				
< 55 yrs	18	13	5	n.s.
≥55 yrs	18	12	6	
**ER expression**				
Negative	14	8	6	n.s.
Positive	22	17	5	
**PgR expression**				
Negative	13	9	4	n.s.
Positive	23	16	7	
**Grading^#^**				
G2	21	17	4	n.s.
G3	15	8	7	
Stage*°				
I-IIA	17	15	2	0.02
IIB-III	16	7	9	
**HER2**				
Negative"	27	21	6	n.s.
Positive"	9	4	5	
**Ki67**				
Negative	22	15	7	n.s.
Positive	14	10	4	
**Treatment response**				
CR+PR	15	11	4	n.s.
SD+PD	21	14	7	

n.s. = not significant; CR = complete response; PR = partial response; SD = stable disease; PD = disease progression. **^#^**According to Elston and Ellis classification (Ref. 31). *According to UICC-TNM classification of malignant tumours, sixth edition 2002 (Ref. 30). °At initial diagnosis time. "IHC 0,1+ and 2+ FISH negative were regarded as negative while IHC 3+ or 2+ FISH positive were regarded as positive.

Conversely, HER2 positive breast tumors appear to be, as expected, less differentiated and of higher stage more frequently than negative ones (Table 3). In accordance with literature data, 6 out of 9 (66.6%) HER2 positive while only 9 out 27 (33.3%) HER2 negative patients respectively responded to docetaxel treatment and this difference was significant (Table 3). Confirmatory results were obtained by student-T test on mean FISH values between responders vs not-responders patients. In fact, responder group showed significantly higher mean FISH values than not-responder (8.53 ± 10.21 vs 2.50 ± 4.12, *p *= 0.027). All HER2-positive patients received trastuzumab in combination with docetaxel while HER2-negative ones were treated with docetaxel with a known influence on and response rate and outcome. To shrink the possible treatment-related bias we test the FISH value difference between docetaxel responders and not-responder in HER2-negative subgroup (n = 27) so removing trastuzumab treatment-related bias. Taking into account the smaller sample size and the lower FISH values (< 2), we found a non-statistically significant difference in mean FISH value with responders patients having higher values (1.64 ± 0.157 vs 1.38 ± 0.146; p = ns). We also performed the same analysis in FISH-positive group (11 pts all receiving docetaxel plus trastuzumab) and we observed also in this small subgroup a similar behaviour (16.86 ± 9.78 vs 9.85 ± 10.53; responders vs not-responders; p = 0.18 ns).

**Table 3:** HER2 expression in relation to main tumor cheracteristics and treatment response

	HER2 expression"			
	
	Total	Low	High	*p *value
**Age**				
< 55 yrs	18	13	5	n.s.
≥55 yrs	18	14	4	
**ER expression**				
Negative	14	10	4	n.s.
Positive	22	17	5	
**PgR expression**				
Negative	13	9	4	n.s.
Positive	23	18	5	
**Grading^#^**				
G2	21	18	3	0.05
G3	15	8	7	
Stage*°				
I-IIA	17	16	1	0.003
IIB-III	16	8	8	
**Ki67**				
Negative	22	18	4	n.s.
Positive	14	9	5	
**Treatment response**				
CR+PR	15	9	6	0.046
SD+PD	21	18	3	

"IHC 0, 1+ and 2+ FISH negative were regarded as negative while IHC 3+ or 2+ FISH positive were regarded as positive. **^#^**According to Elston and Ellis classification (see text for complete reference). *According to UICC-TNM classification of malignant tumours, sixth edition 2002. °At initial diagnosis time. n.s. = not significant; CR = complete response; PR = partial response; SD = stable disease; PD = disease progression.

**Figure 3:**
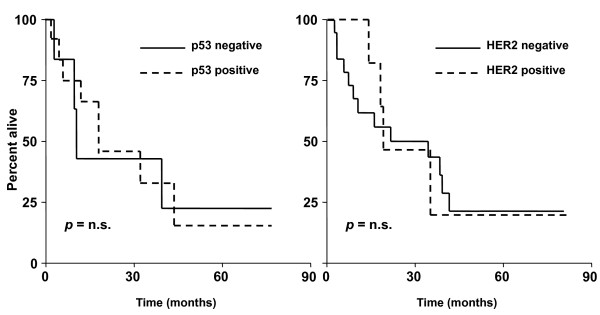
**Kaplan-Meier curves for overall survival according to p53 or HER2 status**. Kaplan-Meier curves for overall survival showed no-significant separation between high vs low-espressors group for both p53 (left panel) and HER2 (right panel). Similar results were obtained for disease-free survival (not shown).

Mean TTP (positive vs negative: 7.9 ± 8.1 vs 9.8 ± 9.4 months; *p *= 0.18 ns) and OS (positive vs negative: 18.1 ± 11.7 vs 21.2 ± 12.1 months; *p *= 0.12 ns) showed a only modest trend towards significance with HER2 positive patients having worse prognosis. Kaplan-Meier survival analysis did not show a significant separation between HER2 positive and negative groups (Figure 3 for OS curves). The same results for both study molecules were obtained even incorporating in responders group patients achieving SD (not shown). Neither HER2 expression nor p53 status were independent predictors of OS and TTS at Cox regression analysis.

Lastly, we also observed at cross-tabulation analysis a clear correlation between HER2 testing with IHC and FISH (*p *= 0.001). Mean ± SD FISH values in negative and positive groups were 1.51 ± 0.223 and 13.09 ± 9.98 respectively.

## Discussion

Some preliminary comments about study limitations will facilitate the discussion of the results. First, presented data originate from a retrospective analysis that is naturally exposed to selection bias. Second, the relative small sample size could reduce the strength of statistical associations and dramatically affects survival analyses. Third, all patients did not receive the same chemotherapy regimen both in term of schedule (weekly or every 3 weeks administrations) and in term of associated drug (5 patient received an association of docetaxel plus capecitabine). Lastly, according to guidelines all HER2 positive patients (both patients that achieve a response and patients who did not) received trastuzumab while negative-ones were treated with docetaxel (alone or in combination). The difference in treatment received and, notably, in the underlying cancer biology makes HER2 positive and negative groups as different populations so affecting our data interpretation.

Within that specific experimental context, IHC-assessed nuclear p53 status failed to show any significant association with outcome and survival parameters. In fact, nuclear expression level of p53 did not differ between responders and not-responders patients. Reasons for this phenomenon cannot be limited to the above mentioned study limitations, probably, should be seek in the mechanisms of action (MoA) of docetaxel and, to a lesser extent, in technical limitations of p53 determination by IHC.

Docetaxel, a semi-synthetic analogue of paclitaxel, is a promoter of microtubule stabilization by direct binding leading to cell cycle arrest at G2/M and apoptosis [33-35]. The β-subunit of the tubulin heterodimer, the key component of cellular microtubules, represent the molecular target of docetaxel [36]. This unique MoA could offer a putative explanation for the lack of association between p53 status and docetaxel sensitivity. In fact, docetaxel is not a direct DNA-damaging drug and docetaxel-induced cell cycle arrest occurs in a late phase of cell cycle (G2/M transition).

Conversely, p53 mainly (but not exclusively) acts in early phases of cell cycle inducing, after DNA damage, a G1 arrest by transactivation of p21Waf1/Cip1, a cyclin-dependent kinase inhibitor [37-39]. Therefore, the subcellular localization of docetaxel molecular target and the timing of docetaxel action during cell cycle do not overlap with those of p53 and this could explain, at least in part, our negative results. Some opposite data were published some years ago about a possible predictive role of *TP53 *mutation on paclitaxel sensitivity in breast cancer [22,23]; Johnson et al [23] proposed a model in which the loss of p53 function reduced the G1 block thus enhancing the efficacy of paclitaxel during mitosis. Our data do not support this hypothesis even accounting for docetaxel over paclitaxel differences.

Lastly, the correlation between p53 nuclear storage measured by IHC and p53 mutation detected by sequencing has been estimated to be less than 75% in breast carcinomas [40]. Indeed, not all mutations yield a stable protein, and some mutations lead to an abnormal protein not detected by IHC. On the other hand, wild-type p53 may accumulate in some tumors as a result of the response to DNA damage, giving a positive IHC result not accounting for *TP53 *mutation [41].

On the other hand, we observed a clear predictive value for HER2 status. Patients with HER2-positive tumors were more likely to respond to docetaxel treatment even taking into account the small sample size. This observation seems to be true independently of patient category (HER2-positive or negative); in fact, in both the whole population and in HER2 subgroups it seems that the higher is the FISH value the higher is the probability to respond to docetaxel. In our opinion, the most likely explanation of our data may resides in the higher proliferation rate of this subset of cancers [25]. Docetaxel, as near-all chemotherapeutic agents, works better in tumors with an higher proliferation index because cancer growth-rate it's "*per se" *the main determinant of cell sensitivity to non-target chemoterapy. Moreover, rapid growth cancers (as HER2 positive breast cancer) have a greater percentage of cells in the M phase of cell cycle and this could represent another element to take into account.

More specific molecular mechanisms, i.e. as for topoisomerase II alpha, are unlikely. In fact, β-tubulin consists of six isotypes, all of which have related aminoacid sequences and are well conserved between species. Class I-βtubulin is the most commonly expressed isotype in human beings, and the most common isotype in cancer cells [42]. The class-I isotype is encoded by the *TUBB *gene located at 6p2513 far from HER2 gene located on chromosome 17. Thus a co-amplification phenomenon is difficult to propose [42].

## Conclusions

FISH-determined HER2 status may predict docetaxel sensitivity in metastatic breast cancer and could be an element to evaluate in the pre-treatment work-up. Obviously, a further prospective validation on a larger sample size is warranted before any possible clinical application. Interestingly, HER2 is a well-known predictor of trastuzumab efficacy and the association of trastuzumab plus taxanes can be considered as a standard of care in the first-line setting, so the possibility to predict treatment response by analysing one parameter (HER2) could be an attractive option.

Conversely, p53 status lack of any significant association with docetaxel sensitivity in the same setting. Probably, TP53 gene mutational analysis could be more informative that IHC, even if a simplistic association between TP53 gene status and taxane treatment response seem to be unlikely given the wide and very complicated molecular pathway related to p53.

## Competing interests

The authors declare that they have no competing interests.

## Authors' contributions

AC: study design, statistical analysis, data interpretation and paper writing; SD: data collection and interpretation; PV: data collection, immunohistochemistry performance and interpretation; OS: data interpretation and paper writing; CP: study design and statistical analysis; GT: data collection and interpretation; CV: data interpretation and paper writing; FDL: data collection, immunohistochemistry performance and interpretation; LM: data collection, immunohistochemistry performance and interpretation; AC: FISH performance and interpretation, data collection; DA: study design, data interpretation and paper writing. All authors read and approved the final manuscript.
